# Parâmetros Ecocardiográficos Simples podem Substituir o Cálculo do Modelo Probabilístico ASCVD?

**DOI:** 10.36660/abc.20220270

**Published:** 2022-05-04

**Authors:** Tonnison de Oliveira Silva, Luiz Eduardo Fonteles Ritt

**Affiliations:** 1 Instituto D’Or de Pesquisa e Ensino Hospital Cardio Pulmonar Salvador BA Brasil Instituto D’Or de Pesquisa e Ensino, Hospital Cardio Pulmonar, Salvador, BA - Brasil; 2 Escola Bahiana de Medicina e Saúde Pública Salvador BA Brasil Escola Bahiana de Medicina e Saúde Pública, Salvador, BA - Brasil

**Keywords:** Doenças Cardiovasculares, Hipertrofia Ventricular, Disfunção Diastólica, Ecocardiografia/métodos, Estratificação de Risco, Hospitalização, Mortalidade

O estudo de Fernandes et al.,^1^ utilizou dados da Coorte ELSA-BRASIL^2 , 3^ *e* demostrou que em pacientes assintomáticos e sem histórico de doença cardiovascular, medidas ecocardiográficas que fazem parte da rotina diária de qualquer serviço de Ecocardiografia, estão associadas de forma independente ao modelo preditor ASCVD. Foram avaliados 2.973 participantes brasileiros sem doença cardiovascular no período de 2008 e 2010. O cálculo do escore ASCVD utilizou dados produzidos em 2008-2010 e 2012-2014, enquanto que a realização da ecocardiografia ocorreu no momento inicial exclusivamente (período 1). Os parâmetros ecocardiográficos com significância estatística após análise de regressão logística multivariada (controlada para índice de massa corpórea, hipertrigliceridemia, atividade física, nível educacional e consumo excessivo de álcool), foram a disfunção diastólica, hipertrofia ventricular esquerda e o volume atrial esquerdo indexado pela área de superfície corpórea. A disfunção diastólica do ventrículo esquerdo foi o preditor com maior força de associação a um elevado risco de eventos cardiovasculares (ASCVD > 7,5%).^1^

A disfunção diastólica é sabidamente um marcador de eventos cardiovasculares, incluindo mortalidade total e hospitalizações por IC.^4^ Na cascata isquêmica a disfunção diastólica, sintomática ou não, é uma das manifestações mais precoces. É útil também na identificação daqueles pacientes no estágio B de insuficiência cardíaca.^4^ O ecocardiograma é a ferramenta não invasiva, disponível e de baixo custo, mais utilizada na avaliação dessa alteração. O grande entrave é que os mecanismos envolvendo a diástole ventricular são complexos, multifatoriais, relacionado a idade, sujeito à alterações hemodinâmicas e de fluxo coronário, tanto agudos e quanto crônicos.^4 - 6^

Expressando o que acabamos de descrever, as diretrizes internacionais focadas nesse tema vem com mudanças significativas ao longo do tempo diminuindo a quantidade de variáveis analisadas, na busca de maior praticidade e acurácia diagnóstica.^5 , 6^ Apesar das modificações já incorporadas, o seu diagnóstico é por vezes difícil, trabalhoso e ainda com resultados “indeterminados”.^4 - 6^ O parâmetro ecocardiográfico mais relevante, encontrado nessa publicação, do ponto de vista de associação com o ASCVD, é talvez o achado ecocardiográficos mais passível de críticas. São até quinze variáveis que podem ser interpretadas quando da verificação da diástole do VE, todos com suas respectivas limitações.^4 - 7^ Isso de certa forma prejudica a sua confiabilidade, reprodutibilidade e por consequente sua validade externa. A HVE foi o segundo achado ecocardiográfico com maior força de associação e por último, o volume do átrio esquerdo.

A hipertrofia ventricular esquerda é um conhecido marcador de eventos cardiovasculares inclusive mortalidade total,^8 , 9^ mas sem um associação definida com desfechos intermediários (escore), como demostrado nesse estudo.

O volume do átrio esquerdo apesar de ser um marcador de função diastólica tem uma fraca relação com a mesma e com as pressões de enchimento do VE,^4^ principalmente nesse perfil específico de participantes analisados. Maiores volumes atriais estão relacionados a maiores pressões nessa cavidade, no entanto esse aumento volumétrico pode ocorrer por outras situações, não diretamente ligadas à doença aterosclerótica. Esse aumento pode advir de uma doença mitral reumática, arritmias atriais e até em modificações fisiológicas vista em atletas saudáveis.^4^

O estudo de Fernandes et al.,^1^ é relevante na medida em que analisou exclusivamente a população brasileira e demostrou associação entre dados do ecocardiograma e o escore probabilístico ASVCD. Então, os parâmetros ecocardiográficos de função diastólica podem substituir o cálculo desse escore preditivo? Apesar de existir uma associação independente entre eles, os sistemas biológicos são complexos e outras variáveis que possam gerar confusão não são contempladas plenamente. Outra questão a ser ponderada é o fato desses três parâmetros serem sujeitos as técnicas variadas de quantificação, podendo sofrer diversas influências e à uma variabilidade inter e intraobservador além do aceitável. No entanto, acreditamos que o estudo de Fernandes et al.,^1^ abre a possibilidade para estudos futuros objetivando avaliar se esses parâmetros anteriormente descritos de fato possam incrementar valor ao ASCVD, refinando sua probabilidade de predição e de sua estratificação de risco.
