# Supplementary material for: Parâmetros Ecocardiográficos Simples podem Substituir o Cálculo do Modelo Probabilístico ASCVD?
Source: Arq Bras Cardiol. 2022 May 4;118(5):925–6. [Article in Portuguese] doi: 10.36660/abc.20220270 (PMC9368864; doi:10.36660/abc.20220270)
Supplement: Translation

# Translation: Can Simple Echocardiographic Parameters Replace The ASCVD Probabilistic Model Calculation?

**Authors:** Tonnison de Oliveira Silva, Luiz Eduardo Fonteles Ritt

**Affiliations:** 1 Brasil Instituto D’Or de Pesquisa e Ensino, Hospital Cardio Pulmonar, Salvador, BA - Brasil; 2 Brasil Escola Bahiana de Medicina e Saúde Pública, Salvador, BA - Brasil

**Keywords:** Cardiovascular Diseases, Hypertrophy, Left Ventricular, Diastolic, Dysfunction, Echocardiography/methods, Risk, Stratification, Hospitalization, Mortality

The study by Fernandes et al.^1^ used data from the ELSA-BRASIL Cohort.^2 , 3^ It showed that in asymptomatic patients with no history of cardiovascular disease, echocardiographic measurements that are part of the daily routine of any echocardiography service are independently associated with the ASCVD predictor model. A total of 2,973 Brazilian participants without cardiovascular disease were evaluated between 2008 and 2010. The ASCVD score calculation used data produced in 2008-2010 and 2012-2014; echocardiography was performed exclusively at the initial moment (period 1). After multivariate logistic regression analysis, the echocardiographic parameters with statistical significance (controlled for body mass index, hypertriglyceridemia, physical activity, educational level and excessive alcohol consumption) were diastolic dysfunction, left ventricular hypertrophy, and left atrial volume indexed by body surface area. Left ventricular diastolic dysfunction was the strongest predictor of association with a high risk of cardiovascular events (ASCVD > 7.5%).^1^

Diastolic dysfunction is a marker of cardiovascular events, including total mortality and hospitalizations for HF.^4^ In the ischemic cascade, diastolic dysfunction, symptomatic or not, is one of the earliest manifestations. It is also useful in identifying those patients with stage B heart failure.^4^ Echocardiography is the non-invasive, available and low-cost tool most used to evaluate this alteration. The major obstacle is that ventricular diastole mechanisms are complex, multifactorial, age-related, and subject to both acute and chronic hemodynamic and coronary flow changes.^4 - 6^

Expressing what we have just described, the international guidelines focused on this topic have reduced the number of variables analyzed with significant changes in the search for greater practicality and diagnostic accuracy.^5 , 6^ Despite the modifications already incorporated, its diagnosis is sometimes difficult, laborious and still with “indeterminate” results.^4 - 6^ The most relevant echocardiographic parameter found in this publication, from the point of view of the association with ASCVD, is perhaps the echocardiographic finding most subject to criticism. There are up to fifteen variables that can be interpreted in verifying LV diastole, all with their respective limitations.^4 - 7^ This somehow impairs its reliability, reproducibility and, consequently, its external validity.

Left ventricular hypertrophy is a known marker of cardiovascular events, including total mortality,^8 , 9^ but without a defined association with intermediate outcomes (score), as demonstrated in this study.

Despite being a marker of diastolic function, the left atrial volume has a weak relationship with it and with LV filling pressures,^4^ especially in this specific profile of analyzed participants. Larger atrial volumes are related to higher pressures in this cavity. However, this volumetric increase can occur due to other situations not directly linked to atherosclerotic disease. This increase may result from rheumatic mitral disease, atrial arrhythmias, and even physiological changes in healthy athletes.^4^

The study by Fernandes et al.^1^ is relevant as it exclusively analyzed the Brazilian population and demonstrated an association between echocardiogram data and the ASVCD probabilistic score. So, can echocardiographic parameters of diastolic function replace the calculation of this predictive score? Although there is an independent association between them, biological systems are complex, and other variables that may confuse are not fully addressed. Another issue to consider is that these three parameters are subject to different quantification techniques and may suffer different influences and an inter- and intra-observer variability beyond acceptable. However, we believe that the study by Fernandes et al.^1^ opens the possibility for future studies aiming to assess whether these parameters described above can add value to ASCVD, refining its prediction probability and risk stratification.
